# Electrochemical fingerprint of street samples for fast on-site screening of cocaine in seized drug powders

**DOI:** 10.1039/c5sc04309c

**Published:** 2016-01-06

**Authors:** Mats de Jong, Nick Sleegers, Jayoung Kim, Filip Van Durme, Nele Samyn, Joseph Wang, Karolien De Wael

**Affiliations:** a AXES Research Group , Chemistry Department , Groenenborgerlaan 171 , 2020 Antwerp , Belgium . Email: karolien.dewael@uantwerpen.be; b Department of Nanoengineering , University California San Diego , CA 92093 , USA; c National Institute for Criminalistics and Criminology (NICC) , Vilvoordsesteenweg 100 , 1120 Brussels , Belgium

## Abstract

We report on a wearable fingertip sensor for on-the-spot identification of cocaine and its cutting agents in street samples. Traditionally, on-site screening is performed by means of colour tests which are difficult to interpret and lack selectivity. By presenting the distinct voltammetric response of cocaine, cutting agents, binary mixtures of cocaine and street samples in solution and powder street samples, we were able to elucidate the electrochemical fingerprint of all these compounds. The new electrochemical concept holds considerable promise as an on-site screening method.

## Abstract 2

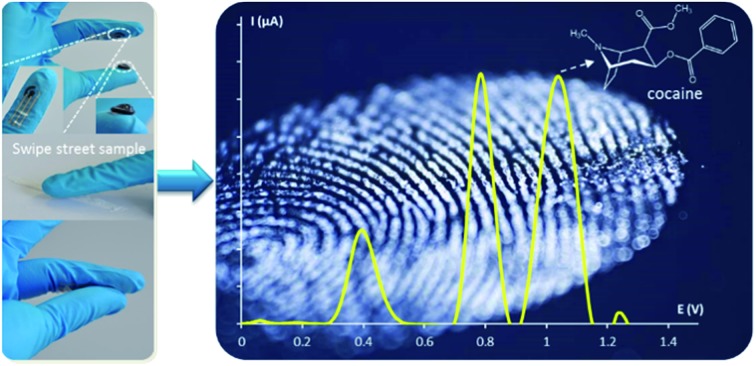
Application of a novel electrochemical fingerprint strategy leads to improved screening, allowing simultaneous detection of cocaine and cutting agents.

## Introduction

Cocaine is one of the most abundant drugs of abuse worldwide, entering different countries mainly *via* airports and harbours, both for local consumption and for distribution.^1^ This alkaloid drug is highly addictive and harmful for people's health.^2^,^3^ In addition to the desired effects for users such as an intense euphoric feeling, undesirable secondary effects like an increased blood pressure, heartbeat rate and respiration rate are experienced.^4^,^5^ After long-term use, the addict becomes tolerant for the desired effects and will experience a state of lethargy, major depression and extreme tiredness when no longer using cocaine.^4^

Customs services at airports and harbours are very keen to monitor passing cargo, luggage and people for the presence of cocaine. On-site screening by means of colour tests based on cobalt thiocyanate is commonly performed. However, these tests are difficult to interpret and not always reliable because of a poor selectivity.^6^,^7^ Usually the colour tests are followed by a confirmation analysis by means of GC-MS (qualitative), GC-FID (quantitative) and HPLC for an unambiguous identification of cocaine and its cutting agents. This confirmation uses a range of laborious and complicated techniques and/or bulky and expensive instruments that should be avoided for routine screenings.^8^ An alternative screening can be performed by FT-IR and Raman spectroscopy, using expensive instrumentation providing spectra that are difficult to interpret without specialized expertise.^9^ To overcome these limitations, the present study aims at a fast, cheap, easy, and selective screening test for on-site detection of cocaine.

**Fig. 1:**
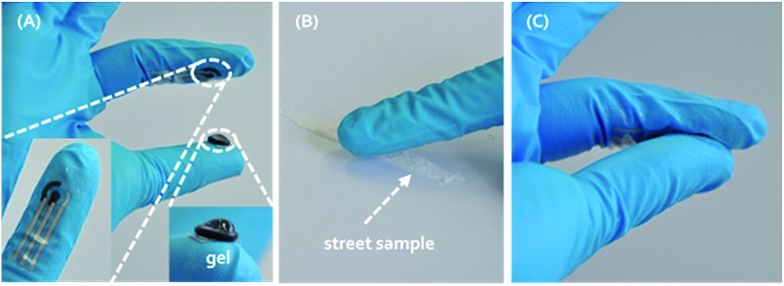
Schematic of the measuring procedure for suspicious powder samples at a wearable fingertip device. (A) The fingertip exhibiting the surface of screen-printed electrode onto a flexible nitrile finger cot (bottom left inset), as well as a conductive gel immobilized upon the thumb (bottom right inset); (B) swiping method of sampling to collect the target powder directly onto the electrode; (C) completion of the electrochemical cell by joining the index finger with electrodes to the thumb coated with the conductive gel electrolyte.

Knowing that cocaine is a redox active molecule,^10^ an electrochemical screening approach by recording voltammograms allows customs to quickly screen unknown powders in cargo and baggage on-site, facilitating high throughput and low cost of detection. Even more, most cutting agents can be sensed along with cocaine because of their redox activity.^11^–^18^ These cutting agents are added to cocaine by the dealers, mainly in order to maximise profit. Typical substances are phenacetin, caffeine, levamisole, lidocaine, paracetamol, procaine, benzocaine, diltiazem, hydroxyzine, boric and benzoic acid, and several sugars like d-sorbitol, myo-inositol, maltose and starch. These compounds all have a white colour like cocaine and some of them, for example lidocaine, paracetamol and phenacetin, have analgesic and anesthetic effects the users will relate to cocaine.^17^,^18^ Voltammograms contain much more information for identification of street samples containing both cocaine and cutting agents compared to the standard colour tests that rely solely on the presence of cocaine. Here we describe the usefulness of electrochemical techniques to measure simultaneously cocaine and its cutting agents in a single voltammetric run *via* square-wave voltammetry (SWV). In order to obtain the full and distinct electrochemical fingerprint of street samples, all cutting agents and cocaine have been electrochemically screened separately in solution and in a binary mixture. Secondly, powder samples were analysed by using the electrochemical approach, allowing a fast and easy on-site detection, without an extensive sample preparation procedure. The sensing device being integrated into a glove/fingertip, allows such on-site detection.^19^ The measuring protocol is presented in Fig. 1. Part (A) represents the finger exhibiting the three electrode surface screen-printed onto a flexible finger cot (bottom left inset), as well as gel immobilized upon a similar substrate (bottom right inset). Part (B) and (C) illustrate, respectively, the ‘swipe’ method of sampling to collect the target powder directly onto the electrode and the completion of the electrochemical cell by joining the index finger with electrodes to the thumb coated with the solid hydrogel electrolyte. The electrochemical fingerprint is recorded in less than a minute by using a rapid square-wave voltammetric approach, giving information on both the presence of cocaine and its cutting agents.

We expect that this new electrochemical fingerprint on a wearable glove platform will greatly enhance the on-site screening of cocaine samples. The ability to rapidly generate this signature with a single scan method using compact, cheap and easy-to-use electrochemical devices should be very useful for on-site detection, performed by customs services. For the first time, the presence of cutting agents in street samples is elucidated in an on-site test.

## Results and discussion

### Voltammetry of pure compounds in solution

**Fig. 2:**
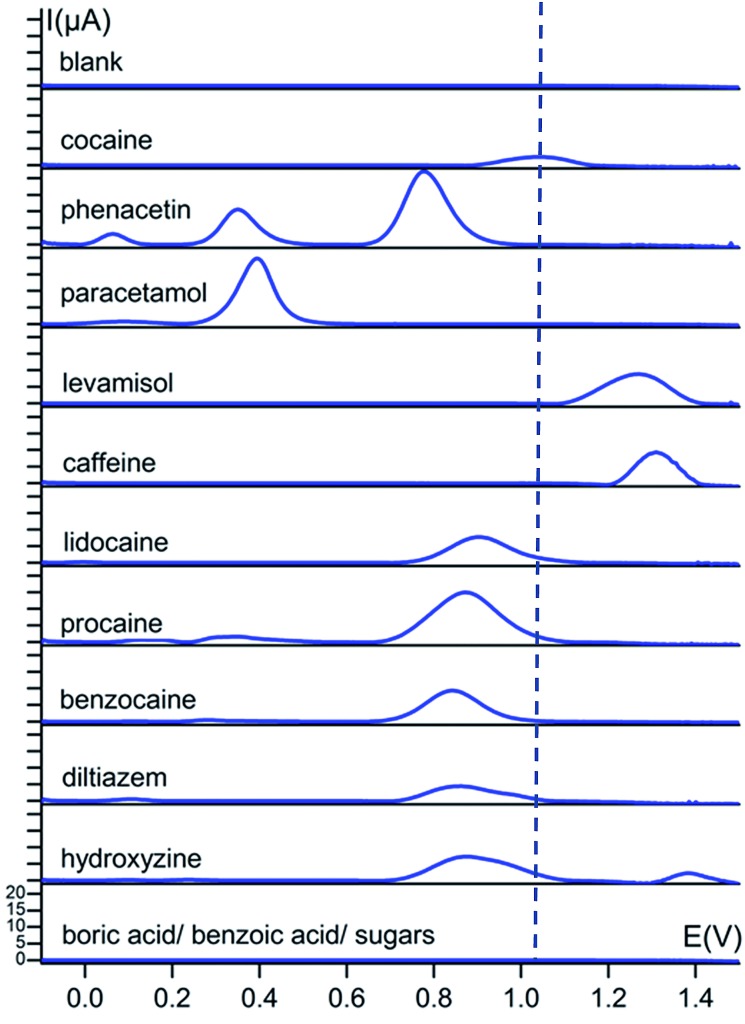
Baseline-corrected square-wave voltammetric responses (*vs.* Ag/AgCl) of 1 mM solutions of cocaine and cutting agents at bare carbon screen-printed electrodes in 0.1 M KCl + 0.020 M KH_2_PO_4_ buffer (pH 7). The dotted line represents the characteristic oxidation potential of cocaine at 1.04 V. The *y*-axis has the same scale in every voltammogram.

**Scheme 1:**
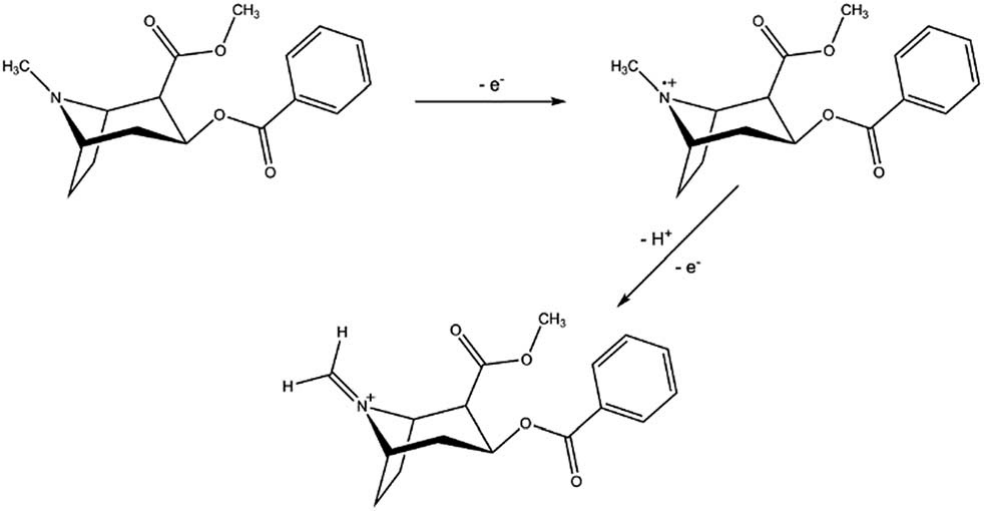
Oxidation process of cocaine at a carbon screen-printed electrode at potential 1.04 V. The second reaction occurs immediately after the formation of the intermediate radical, causing only one visible peak in a square-wave voltammogram.

A first and crucial step is the screening and assessment of the redox behaviour of cocaine and its cutting agents in solution to unravel the electrochemical fingerprint of these compounds. Square-wave voltammograms of 1 mM cocaine or cutting agent in a 0.1 M KCl + 0.020 M KH_2_PO_4_ solution at a bare carbon screen-printed electrode are shown in Fig. 2, corrected for the background current by using the moving average principle, integrated in the NOVA 1.11 software. The 1 mM cocaine solution exhibits a weak oxidation process at 1.04 V which can be contributed to the oxidation of the tertiary amine group present in the hexagon structure of cocaine as is shown in Scheme 1.^10^ Most cocaine samples are cut with different substances; identification of these cutting agents might be of interest from a toxicological point of view. The parameters of the used square-wave voltammetry procedure were optimized prior the analysis and are described in the experimental part.

Cutting agents benzoic acid and boric acid show no redox activity over the studied potential range, which corresponds with the literature, only showing activity at potentials below –1 V for these compounds.^14^,^15^ The sugars d-sorbitol, maltose, starch and myo-inositol also show no redox activity in the studied potential range. Fig. 2 illustrates that the cocaine signal at 1.04 V is rather isolated from the other characteristic signals of the cutting agents. The closest more positive process is the peak of levamisole at 1.27 V and the closest more negative is the peak of lidocaine at 0.90 V and its analogues procaine and benzocaine, 0.87 V and 0.84 V, respectively. All these oxidation processes occur at tertiary (levamisole and lidocaine) and primary (benzocaine and procaine) amine functional groups.^11^,^12^,^17^,^20^ Apart from these peak potential values, the number of characteristic peaks as well as the onset potentials give useful information on determining their fingerprint. Phenacetin, for instance, displays three definitive redox processes, so even if there is an overlap with the primary oxidation peak, the present cutting agent can still be identified by its secondary or tertiary signal. The primary signal at 0.77 V results from the irreversible oxidation of phenacetin to *N*-acetyl-*p*-benzoquinone imine (NAPQI). The smaller secondary peaks at 0.06 and 0.35 V are a result of the oxidation of 4-aminophenol and NAPQI, respectively.^21^ These two secondary peaks are also present for paracetamol and are caused by the same compounds.^18^ Hydroxyzine and diltiazem both show an oxidation peak at 0.87 V, caused by the oxidation of a free –OH group and a tertiary amine, respectively.^16^,^22^ Caffeine has the highest oxidation potential at 1.31 V, resulting from the oxidation of the compound to a 4,5-diol analogue.^13^

This extensive screening and rich information content leads to a distinct electrochemical fingerprint of cocaine and its cutting agents that can serve as a powerful reference when the presence of cocaine in an unknown sample needs to be confirmed or ruled out.

The applied conditioning step at 1.5 V (as is described in the experimental section) was performed to extract maximal information from our substances. For instance, phenacetin would only show a single peak at 0.77 V instead of three peaks if no conditioning step was performed. Due to the conditioning step, redox processes related to NAPQI and 4-aminophenol take place, enriching the fingerprint of the samples.

The limit of detection (LOD) for pure cocaine on the non-modified screen-printed electrode (SPE) surface in solution was determined at 2 μM. This corresponds to a quantity of 34 ng on the electrode surface. The LOD was determined based on the standard deviation of the intercept and the average slope of the linear calibration curves that were obtained.

This LOD is considerably lower compared to the colour screening tests (13.8 μM) and the accredited GC-MS technique used at the National Institute of Criminalistics and Criminology (NICC) of Belgium (18.4 μM).

### Voltammetry of binary mixtures in solution

An important second step is the assessment of the redox behaviour of cocaine and the cutting agents in mixtures to elucidate potential masking (overlapping peak) phenomena.

**Fig. 3:**
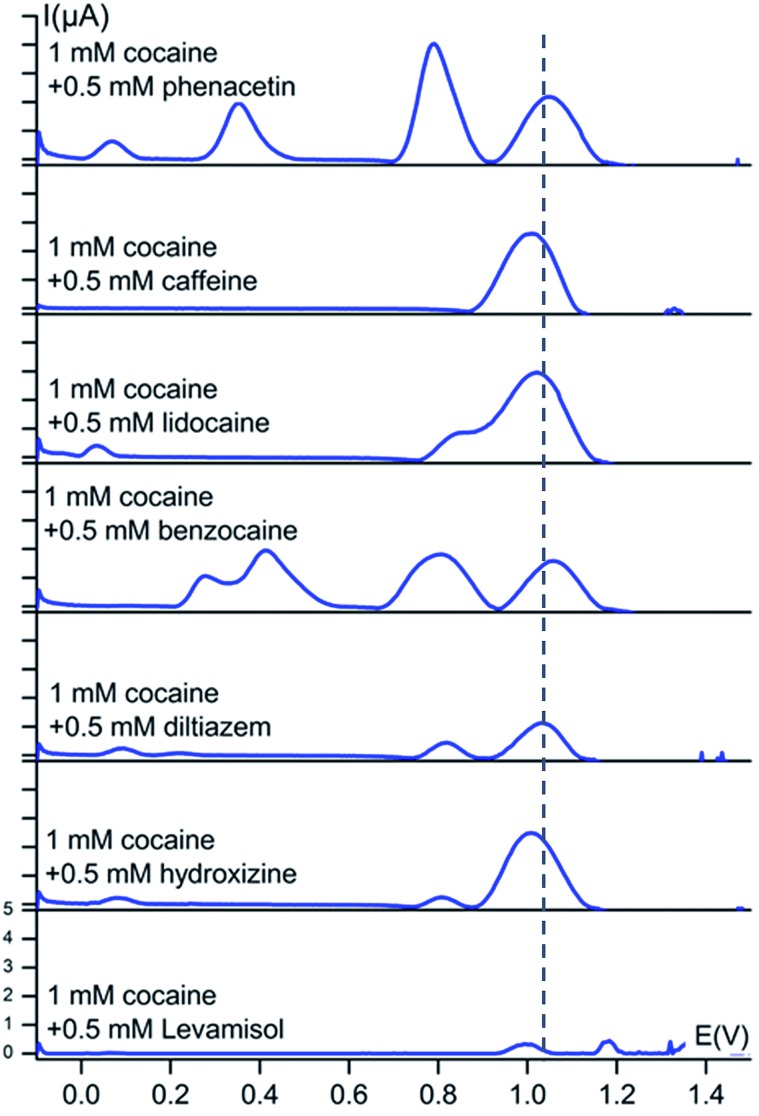
Baseline-corrected square-wave voltammetric responses (*vs.* Ag/AgCl) of 1 mM solutions of cocaine with 0.5 mM cutting agent at bare carbon screen-printed electrodes in 0.1 M KCl + 0.020 M KH_2_PO_4_ buffer (pH 7). The dotted line represents the characteristic oxidation potential of cocaine at 1.04 V. The *y*-axis has the same scale in every voltammogram.

Square-wave voltammograms of 1 mM cocaine and 0.5 mM cutting agent in a 0.1 M KCl + 0.020 M KH_2_PO_4_ solution (pH 7) at a bare carbon screen-printed electrode are shown in Fig. 3.

There are no significant peak shifts visible for the cocaine signal and the cutting agent peaks do occur at their characteristic potential values. This peak potential of the cocaine oxidation process is only slightly shifted in the presence of benzocaine, levamisole, caffeine, phenacetin, lidocaine, diltiazem and hydroxyzine with a value of +18 mV, –12 mV, –32 mV, +13 mV, –17 mV, –7 mV and –32 mV respectively. As is clear from Fig. 2, the differences between the oxidation potential of cocaine and the redox potential of the cutting agents are much bigger compared to the small shifts observed when analysing mixtures. Benzocaine does, however, show more complex behaviour in mixture with cocaine. Although the mixture shows both the characteristic peaks of cocaine and benzocaine, the voltammogram reveals an additional set of peaks in the 0.25–0.45 V range. These processes are not observed when the individual components are measured separately and, therefore, it results from a certain interaction between cocaine and benzocaine, which is unknown at this point. However, the set of peaks is well isolated from the cocaine signal itself and, therefore, provides additional information about the presence of cocaine and benzocaine in a mixture. Minor secondary peaks are also visible at lower potentials for lidocaine, diltiazem and hydroxyzine, when in mixture with cocaine, and are thus useful for the assessment of the redox processes in the mixtures.

Fig. 3 shows a partial overlap of the cocaine oxidation signal with the lidocaine oxidation signal. This slight overlap does not result in a problem for cocaine detection. The used concentration of lidocaine in this section, relative to cocaine, is much higher than the average content of lidocaine found in seized cocaine street samples. In 2014 and 2015, 13.5% of the seized cocaine samples, analysed by NICC using accredited GC-MS and GC-FID techniques, contained lidocaine with an average concentration of 5 wt%.

It is also clear from Fig. 3 that the signal intensity for cocaine drops significantly when in mixture with levamisole. This observation also applies to the levamisole signal. At this point, it is unclear what causes this decrease in intensity for these components. Most importantly, cocaine could still be easily detected. Since we develop a screening technique without quantification, the decreased intensity forms no problem for the application. The levamisole content used in the experiments was once again higher than the average content of levamisole in seized cocaine street samples. In 2014 and 2015, 57% of the seized cocaine samples, analysed by NICC using accredited GC-MS and GC-FID techniques, contained levamisole with an average concentration of 12 wt%.

### Voltammetry of street samples in solution

**Fig. 4:**
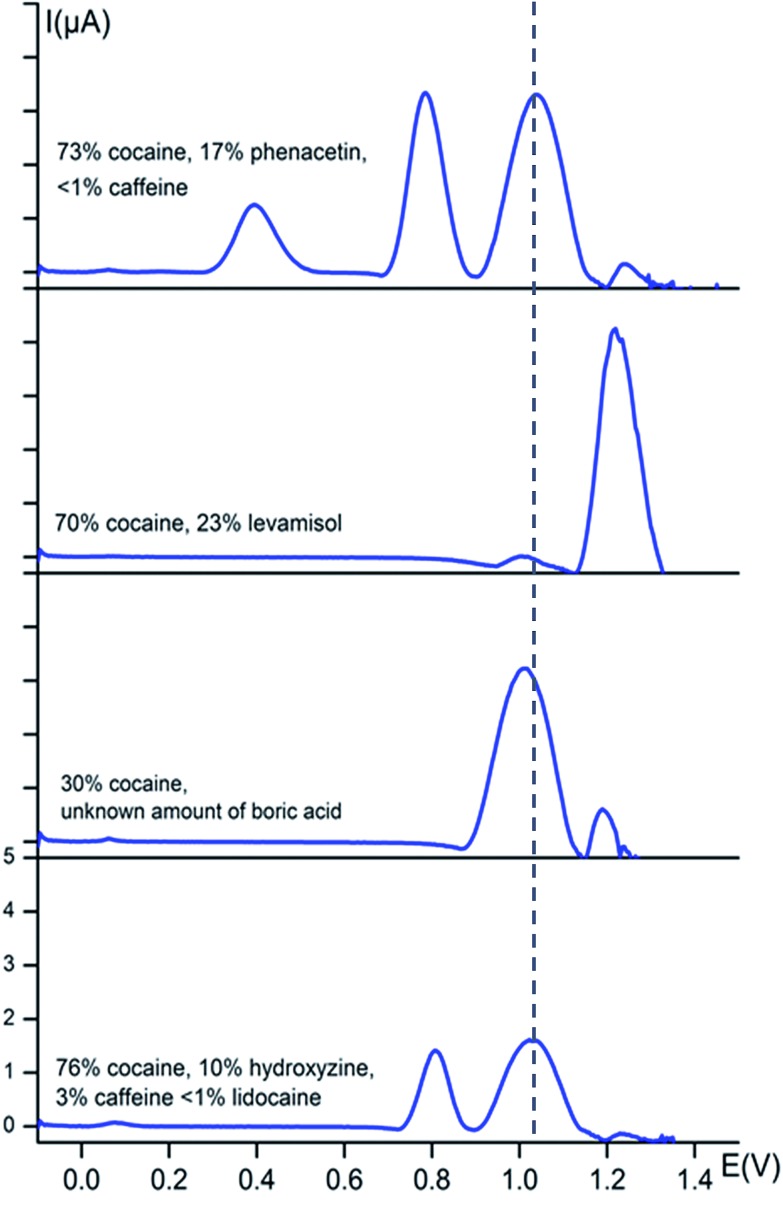
Baseline-corrected square-wave voltammetric responses (*vs.* Ag/AgCl) of street samples at bare carbon screen-printed electrodes in 0.1 M KCl + 0.020 M KH_2_PO_4_ buffer solution (pH 7). The dotted line represents the characteristic oxidation potential of cocaine at 1.04 V. The *y*-axis has the same scale in every voltammogram. Each solution was prepared in such way that it contains 1 mM of cocaine.

In order to test the potential of the fingerprint approach for the detection of cocaine in street samples, square-wave voltammograms of several seized street samples were recorded in buffer solution (0.1 M KCl + 0.020 M KH_2_PO_4_ at pH 7) and corrected for the background current as done before. All solutions were made such that they all contained 1 mM of cocaine and the corresponding voltammograms are shown in Fig. 4. Before the analysis, the qualitative and quantitative composition of the street samples was determined at the National Institute of Criminalistics and Criminology (NICC) using accredited GC-MS and GC-FID methods.

Fig. 4 shows that the cocaine peak is clearly detectable in street samples containing phenacetin, caffeine, levamisole, boric acid, hydroxyzine and lidocaine, even when the cocaine amount is only 30 wt% as is the case in the third sample. Knowing that NICC in Belgium found that 95% of all seized cocaine samples in 2014 contained more than 35 wt% of cocaine, this method is very promising to explore further for fast on-site screening. Even more, based on the observations made in Fig. 2 and 3, the presence of the cutting agents could be evidenced.

Two of the characteristic phenacetin peaks are clear and sharp in the first sample at their characteristic potentials of 0.77 and 0.35 V, whereas the signal for levamisole is also clearly visible at its characteristic potential of 1.27 V in the second sample. The second sample clearly shows the redox processes of cocaine and levamisole at their fingerprint potentials, *i.e.* 1.04 and 1.27 V, respectively. The percentage cocaine is *ca.* 30% in the third sample, however, clearly visible in the square-wave voltammogram. In the fourth sample we see a peak at approximately 0.80 V, which is characteristic for hydroxyzine. Also a very small wave is visible at 0.10 V which is characteristic for either hydroxyzine or diltiazem. A small peak is also visible at approximately 1.30 V, which indicates the presence of caffeine. We were not able to detect the signal for lidocaine in this particular street sample, but this can be explained by the very low concentration of the compound in the sample (<1%).

### Voltammetry of powder street samples

For easy on-site use, it is appropriate to detect the samples directly in its powder form, rather than in solution, to avoid sample preparation. In order to do so, we use the on-site sweeping method, as is explained in detail in the experimental section. A conducting gelatine hydrogel was used as an electrolyte for the electrochemical measurements.

**Fig. 5:**
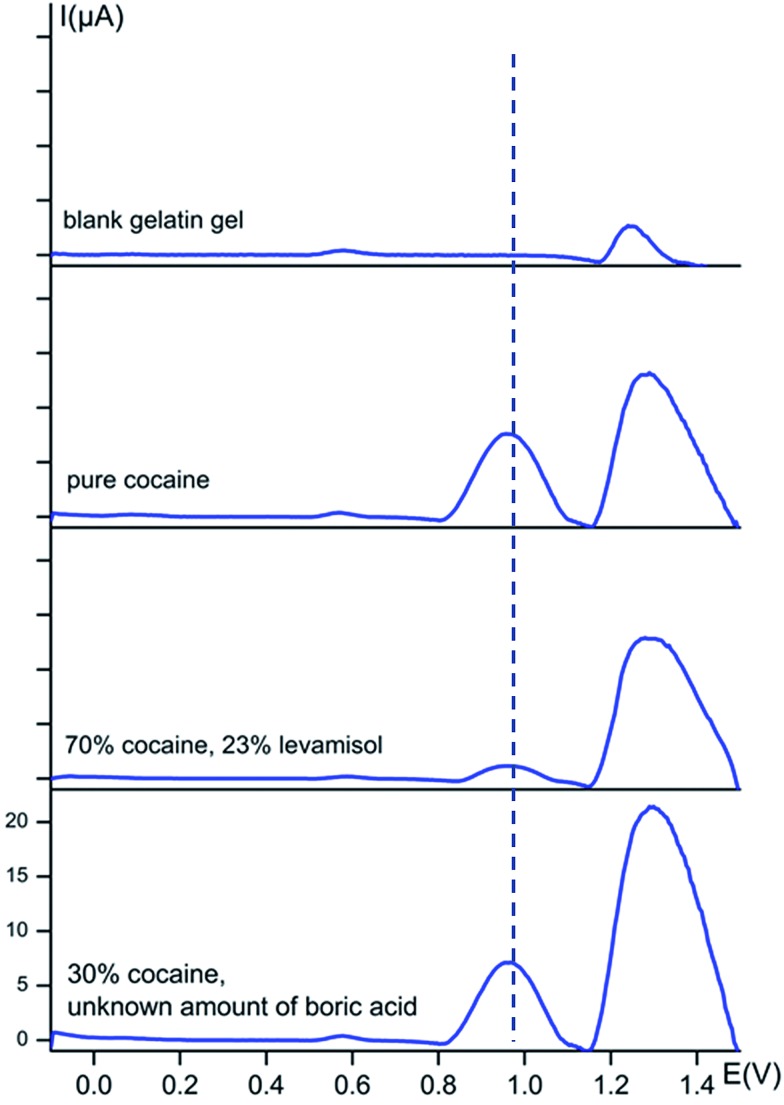
Baseline-corrected square-wave voltammetric responses (*vs.* Ag/AgCl) of powder street samples at bare carbon screen-printed electrodes using a gelatin gel B hydrogel containing 0.1 M KCl + 0.020 M KH_2_PO_4_ buffer solution (pH 7) as electrolyte. The dotted line represents the oxidation potential of cocaine at 0.97 V. The *y*-axis has the same scale in every voltammogram.

Square-wave voltammograms of several seized street samples were recorded and presented in Fig. 5, as well as a blank to retain the response of the gelatine gel itself and a sample only containing pure cocaine. A conditioning step was performed at 0 V rather than the previous chosen 1.5 V in solution, to optimize the sensitivity towards cocaine.

Clearly, the gel itself causes an intense electrochemical signal around 1.30 V and a small peak at 0.57 V. Any information at *ca.* 1.30 V will, therefore, not be applicable to a certain cutting agent. However, it is clear that this method works incredibly well for rapid on-site cocaine detection. The cocaine peak is very intense when pure cocaine is measured, as is clear from the second voltammogram in Fig. 5. The cocaine peak is also well detectable for the street sample containing 23% levamisole and for the sample with only 30% cocaine and an unknown amount of boric acid. A small shift in the peak potentials is visible for powders entrapped in the gelatine matrix compared to the solutions. The cocaine peak is now situated at a potential of 0.97 V instead of 1.04 V.

This information-rich and single run square-wave voltammetry method, using electrodes immobilized on the fingertip of a glove, holds considerable promise for the fast on-site screening of suspicious cargo and persons on the presence of cocaine and its cutting agents.

### Reproducibility

The reproducibility of the obtained signals in solution and powders was studied by performing five measurements, with each time a different SPE, on several of the earlier studied samples, all containing 1 mM cocaine. In solution we obtained results concerning the peak potential of cocaine for pure cocaine and three street samples. A pure cocaine solution showed the characteristic peak at 1.04 ± 0.02 V, while for a first street sample containing 73% cocaine and 17% phenacetin, a second street sample containing 76% cocaine, 3% caffeine, 10% hydroxyzine and less than 1% lidocaine and a third street sample containing 30% cocaine and an unknown amount of boric acid showed peak potentials of 1.06 ± 0.01 V, 1.03 ± 0.01 V and 1.03 ± 0.01 V respectively.

The results show a maximal peak potential standard deviation of ±20 mV, likely caused by small differences in the internal reference electrodes between different SPE's. The potential shifts are, however, small and don't cause any problems to identify cocaine, also because these small shifts occur for the cutting agents as well.

In powder form, the same experiment was performed for pure cocaine, using the conductive gel as electrolyte and the obtained peak potential value was 0.97 ± 0.01 V, showing the robustness and reproducibility of the approach. This stability was assured by the rubber ring, immobilized on the thumb of the fingertip device as is described in the experimental section. This ring keeps the conductive gel in place and it guarantees a constant applied force on the electrode surface while both fingers are joined during the experiments, leading to stable baselines. The small potential variation also shows that swiping the electrode surface over the powders doesn't lead to a different electrochemical response caused by damaged electrode surfaces. This is one of the reasons why graphite electrodes were used.

## Experimental

### Reagents and materials

The cocaine·HCl standard was purchased from Lipomed (Arlesheim, Switzerland). Standards of phenacetin, diltiazem, lidocaine, procaine, hydroxyzine, benzocaine, paracetamol and myo-inositol were purchased from Sigma-Aldrich (Diegem, Belgium). Standards of benzoic acid and levamisole were purchased from Acros Organics (Geel, Belgium). Standards of caffeine, boric acid, glucose, maltose and starch were purchased from VWR Chemicals (Leuven, Belgium) and a standard of d-sorbitol was purchased from Merck Chemicals KGaA (Overijse, Belgium). Authentic cocaine street samples were obtained from the National Institute for Criminalistics and Criminology (Brussels, Belgium). Gelatine gel B was supplied by PB gelatins (United Kingdom). Carbon ItalSens IS-C Screen Printed Electrodes (SPE) were purchased from PalmSens (Utrecht, The Netherlands) and were used during all electrochemical measurements. The electrode surface area is 7.07 mm^2^. All laboratorium-based electrochemical measurements were performed using a Metrohm μAutolab III Potentiostat and NOVA 1.11 software.

### Synthesis of the conducting gel

A mixture of 2.5 wt% gelatine gel B in a 100 mM KCl and 20 mM KH_2_PO_4_ buffer was heated in an Eppendorf tube to 50 °C for 15 minutes followed by additional mixing until the solution became homogeneous. The solution was then transferred to a syringe in which the hydrogel was formed and aged during the next 16 hours at room temperature before use in the experiments.

### Determination of samples in solution

The buffer solution used for the experiments was optimized to obtain maximal peak separation and sensitivity. The 0.1 M KCl + 0.020 M KH_2_PO_4_ buffer allowed the identification of cocaine without overlapping the signals of the cutting agents, as is shown in Fig. 2. Solutions of 1 mM cocaine and all cutting agents were prepared in 0.1 M KCl + 0.02 M KH_2_PO_4_ buffer (pH 7) and stored at 4 °C before analysis. Each solution was analyzed separately by putting a 50 μL drop on the SPE and square-wave voltammetry was performed in order to detect characteristic redox processes for each substance. This was also performed for binary mixtures of cocaine and a cutting agent as well as for authentic street samples.

### On-site swiping method

Suspicious powders were analysed directly by using a gelatine gel as a solid electrolyte. The SPE was inserted in a small SPE connector (with wires connected to the potentiostat) which was fixed on a metal ring. This ring is worn by the executer of the experiment on the index finger of a nitrile glove. The SPE was swiped gently over the suspicious powder in a manner which allowed some of the powder to be transferred onto the working electrode of the SPE. 100 μL of the conductive gel was added with a syringe to a small reservoir on the thumb, surrounded by a small rubber ring. The rubber ring keeps the gel in place and was immobilized on the thumb using cyanoacrylate glue.

Both fingers were joined together, leading to completion of the electrochemical cell where upon the electrochemical measurement could be started. Each electrode and portion of gel was used only once.

### Square-wave voltammetry

Square-wave voltammetry (SWV) was performed to characterize the electrochemical fingerprint of cocaine (street) samples. A conditioning potential of 1.5 V was applied for 5 s, followed by a second conditioning step of 5 s at 0 V before a scan from –0.1 V to a final potential of 1.5 V *vs.* Ag/AgCl was performed. All scans were performed at a frequency of 10 Hz, with an amplitude of 25 mV, and a step potential of 5 mV. For the experiments with powders instead of solution, a single conditioning step of 5 s at 0 V was performed.

### Moving average iterative background correction

A baseline correction method was built in the SWV procedure in the NOVA 1.11 software to automatically correct for the raising background current in order to make the voltammograms easier to interpret. In brief, the method compares the value of a data point *A*_*i*_ to the values of the previous and next data points *A*_*i*–1_ and *A*_*i*+1_. If the value of data point *A*_*i*_ is higher than the average of the values of points *A*_*i*–1_ and *A*_*i*+1_ (as is the case for an oxidation peak), the average of the values of *A*_*i*–1_ and *A*_*i*+1_ will replace the value of *A*_*i*_ to construct the corrected baseline. In all other cases when *A*_*i*_ is lower or the same as the average of *A*_*i*–1_ and *A*_*i*+1_, *A*_*i*_ will be the value used for the corrected baseline. This process was performed for each two data points in the voltammogram and repeated until the value of *A*_*i*_ never exceeds the average of the values of *A*_*i*–1_ and *A*_*i*+1_ anymore, with a maximum of 1000 iterations. The corrected baseline is now assembled and the background current will be zero. Positive currents are only visible at peaks of oxidation processes.

## Conclusions

We have demonstrated the applicability of an electrochemical fingerprint approach to identify cocaine and its cutting agents in street samples. Direct analysis with minimal sample preparation is possible thanks to the integration of the screen-printed electrode in a glove and by using a conductive, flexible gelatine hydrogel as electrolyte. This new approach allows rapid on-site detection at points of interest such as airports and harbours, by simply swiping the electrode system over the suspicious powder, join both fingers and start the square-wave voltammetric measurement. Determination of the distinct electrochemical fingerprint of both cocaine and the cutting agents in solution provides all necessary analytical information to detect cocaine and the cutting agents on-site in unknown suspicious samples. The new concept thus holds considerable promise as a portable, on-site applicable screening method aimed at the rapid identification of cocaine/drugs (street) samples. To achieve this, a switch from a big potentiostat (like μAutolab III) to a miniaturized, portable potentiostat device should be made. With the rapid development of wireless systems and communication, the transmission of results to a smartphone or a tablet would be of substantial utility to on-site drug investigations.
